# The effects of Pycnogenol, a pine bark extract on pulmonary inflammation by Asian sand dust in mice

**DOI:** 10.17221/77/2023-VETMED

**Published:** 2024-01-24

**Authors:** So-Won Pak, Se-Jin Lee, Woong-Il Kim, Yea-Gin Yang, Young-Kwon Cho, Joong-Sun Kim, Tae-Won Kim, Je-Won Ko, Jong-Choon Kim, Sung-Hwan Kim, In-Sik Shin

**Affiliations:** ^1^College of Veterinary Medicine and BK21 FOUR Program, Chonnam National University, Buk-gu, Gwangju, Republic of Korea; ^2^College of Health Sciences, Cheongju University, Cheongju-si, Chungbuk, Republic of Korea; ^3^BK21 FOUR Program, College of Veterinary Medicine, Chungnam National University, Daejeon, Republic of Korea; ^4^Jeonbuk Branch, Korea Institute of Toxicology (KIT), Jeongeup-si, Jeonbuk, Republic of Korea

**Keywords:** airway inflammation, fine dust, MMP-9, *Pinus pinaster Aiton*

## Abstract

Asian sand dust (ASD), also called China dust or yellow dust, mainly occurs in East Asia during spring and autumn. Because ASD enters the body mainly through the respiratory system, it can cause respiratory disorders or worsen underlying diseases. Because of this, it has become an important health concern that threatens the well-being of humans and animals. In this study, we investigated the effects of 15 and 30 mg/kg of Pycnogenol (PYC15 and 30 groups), a pine bark extract, on ASD-induced pulmonary inflammation in mice. We evaluated the inflammatory cell counts, inflammatory cytokines, and matrix-metalloproteinase (MMP)-9 expression in animal models. PYC administration significantly decreased inflammatory cell infiltration into lung tissue; this was accompanied by a reduction in the levels of proinflammatory mediators including interleukin (IL)-1β (*P* **<** 0.01), IL-6 (*P* **<** 0.01) and tumour necrosis factor-α (*P* **<** 0.01) in bronchoalveolar lavage fluids of ASD-exposed mice (ASD group). Histological analysis revealed that PYC suppressed ASD-induced pulmonary inflammation. Moreover, PYC suppressed the levels of matrix-metalloproteinase (MMP)-9 in the lung tissue of ASD-exposed mice, indicating that PYC reduced ASD-induced pulmonary inflammation by suppressing MMP-9. Together, these results indicate that PYC as the potential to treat ASD-driven pulmonary inflammation.

Asian sand dust (ASD) gradually emerges as an important health concern because it threatens the well-being of humans and animals (Roy et al. 2023). Also known as the China dust or yellow dust, ASD forms in the Gobi deserts of China and Mongolia (Tan et al. 2017). It mainly occurs during spring or autumn and is carried by air currents to East Asia countries including Korea and Japan (Hashizume et al. 2020). ASD also contains various harmful components including particulate matter, heavy metals, organic compounds, mycotoxins, and allergens, which threaten the health of humans and animals (Dasari et al. 2020; Hasunuma et al. 2021). Exposure to ASD may contribute to the development of respiratory diseases or exacerbate underlying respiratory conditions through the production of inflammatory mediators like cytokines, chemokines, and reactive oxygen species (Kim et al. 2023). However, the development of therapeutic agents for the treatment of ASD-induced respiratory disorders has been very slow.

Pycnogenol (PYC), a standardised extract of French maritime pine bark (*Pinus pinaster Aiton*), contains several active components, including proanthocyanins. PYC, which is used worldwide as an herbal remedy and as a nutritional and dietary supplement for the management of various disorders including inflammatory and circulatory diseases, has antioxidant, anti-inflammatory, anticancer, and antimicrobial effects (Torras et al. 2005; Yang et al. 2014; Hamed et al. 2022; Nattagh-Eshtivani et al. 2022). PYC is also used to manage respiratory diseases like asthma, rhinitis, pulmonary fibrosis, and chronic obstructive pulmonary disease (Wilson et al. 2010; Liu et al. 2016; Shin et al. 2016; Park et al. 2022). These previous studies indicate that PYC may have therapeutic potential to treat inflammatory pulmonary diseases. Here, we hypothesised that PYC may effectively inhibit ASD-driven pulmonary inflammation.

To test this possibility, we investigated the therapeutic effects of PYC on a model of ASD-induced pulmonary inflammation. To determine the effect of PYC, we analysed inflammatory cell infiltration, inflammatory cytokines, histological changes, and the expression of the inflammation-related protein on lung tissue.

## MATERIAL AND METHODS

### Physicochemical characterization of Asian sand dust

ASD was purchased from Power Technology Inc. (Arden Hills, MN, USA) and was made of 50% JIS Z8901 Class 8 (SiO_2_, Al_2_O_3_, MgO, Fe_2_O_3_, TiO_2_, CaO) and 50% natural SiO_2_. ASD’s morphology and size were determined using scanning electron microscopy (SEM) on Zeiss Gemini500 scanning electron microscope (Carl Zeiss Meditec AG, Jena, Germany) and transmission electron microscope (TEM) on a JEM-2100F electron microscope (JEOL, Tokyo, Japan). ASD’s purity was determined using energy-dispersive X-ray spectroscopy using a Zeiss Gemini500 scanning electron microscope equipped with X-Max^N^ 150 mm^2^ silicon drift detector (Oxford Instrument, Abingdon, UK).

### Ethical declaration

Ethical approval for developing the ASD-induced pulmonary inflammation model was granted by the Institutional Animal Care and Use Committee of Chonnam National University (Approval No. CNU IACUC-YB-2022-114).

### Experimental animals and development of the ASD-induced pulmonary inflammation model

The ASD-induced pulmonary inflammation model was developed as described previously (Kim et al. 2023). PYC was purchased from Horphag Research Ltd. (Le Sen, France). PYC contains a wide variety of procyanidins that range from monomeric catechin (0.15 mg/g, 0.89%) and taxifolin (0.17 mg/g, 0.21%). Male C57BL/6 mice (6 weeks old) were purchased from Samtako (Osan, Republic of Korea) and divided into the normal control (NC), the ASD, and PYC (PYC15 and 30) groups. ASD (40 mg/kg) was administered to the ASD and PYC groups via intranasal instillation on days 1, 3, and 5 under mild anaesthesia. PYC was administered to the PYC groups daily from day 1 to day 6 using oral gavage. Mice were sacrificed 48 h after the final ASD instillation, followed by tracheostomy to obtain bronchoalveolar lavage fluids (BALF) as described previously (Jung et al. 2020). Briefly, after endotracheal tube insertion into the incised trachea, the lung was lavaged twice with 0.7 ml of cold phosphate-buffered saline (PBS, total volume: 1.4 ml), which was then centrifuged. The BALF supernatants were then used to assess the levels of interleukin (IL)-1β, IL-6 and tumour necrosis factor (TNF)-α using commercial enzyme-linked immunosorbent assay kits (BD Biosciences, San Jose, CA, USA). The BALF pellets were resuspended in PBS and the number of total inflammatory cells was counted using a cell counter (Thermo Fisher Scientific, San Diego, CA, USA). To determine the number of differential inflammatory cells in BALF, the pellets resuspended in PBS were attached on glass slides using cytospin and then stained with Diff-Quik reagent (Sysmex, Kobe, Japan). The numbers of inflammatory cells, including neutrophils, macrophages, eosinophils, and lymphocytes were then counted under a light microscope, and the ratio of differential cells out of the total number of inflammatory cells was determined.

### Western blot

After BALF sampling, the right lung was homogenised, and the total protein of each sample was determined using the Bradford reagent (Sigma-Aldrich, St. Louis, MO, USA). Western blot analysis was done as described previously (Jung et al. 2020) using primary antibodies against matrix-metalloproteinase 9 (MMP-9, Cell Signalling, Danvers, MA, USA) and β-actin (Cell Signalling, dilution 1 : 1 000). Protein signals were detected using ChemiDoc (Bio-Rad, Hercules, CA, USA).

### Histological analysis of lung tissue

After collecting BALF, left lung tissues were fixed with paraformaldehyde and then embedded on paraffin blocks. They were then sectioned at 4 μm and stained using haematoxylin and eosin (Sigma Aldrich, St. Louis, MO, USA) to determine the effects of PYC on ASD-induced pulmonary inflammation. To assess the effect of PYC on the expression of MMP-9 (Cell Signalling), Nrf2 (Cell Signalling), and 8-OHdG (Cell Signalling) in lung tissue, we used immunohistochemistry (Vector Laboratories, Burlingame, CA, USA). Quantitative analysis of the inflammatory responses was determined using IMT i-Solution, an image analyser (Vancouver, BC, Canada).

### Statistical analysis

Data are presented as means ± standard deviation (SD). Statistical significance was determined by analysis of variance followed by multiple comparison tests with Dunnett’s adjustment. *P* < 0.05 and *P* < 0.01 indicate statistically significant differences.

## RESULTS

### Physiochemical characterization of ASD

**Figure 1:**
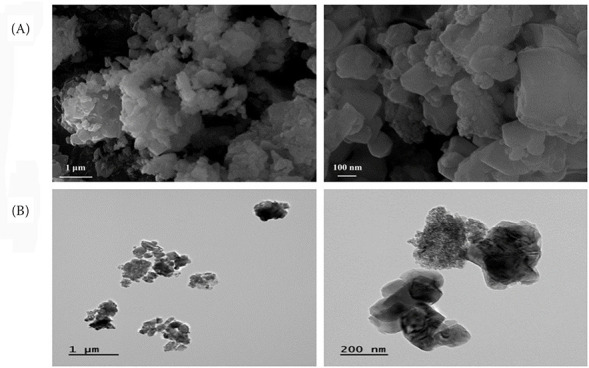
Morphology of Asian sand dust (ASD) (A,B) ASD morphology was determined using scanning electron microscopy (A) and transmission electron microscopy (B)

SEM and TEM analyses revealed that the morphology of ASD was mostly spherical (Figure 1A,B). Energy-dispersive X-ray spectroscopy revealed that the ASD contained O (44.19%), Si (47.53%), Al (4.86%), Fe (2.25%), Mg (0.68%), Ca (0.26%), and Ti (0.23%).

### Effects of PYC on the number of inflammatory cells in BALF from the ASD-induced model of pulmonary inflammation

**Figure 2:**
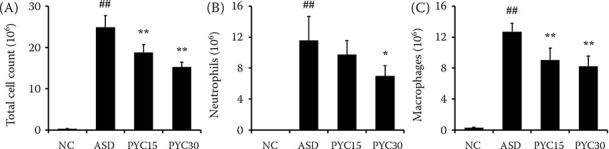
Effects of PYC on inflammatory cell counts in the BALF of ASD-exposed mice (A) Total cell count, (B) neutrophils, (C) macrophages ASD = ASD intranasal instillation + PBS treatment; NC = PBS intranasal instillation + PBS treatment; PYC 15 and 30 = ASD intranasal instillation + PYC treatment at 15 mg/kg and 30 mg/kg, respectively Data are presented as mean ± SD (*n* = 5); ^##^*P* < 0.01 vs NC, *^,^**P < 0.05 and < 0.01 (vs ASD), respectively

BALF from the ASD group had markedly higher total cell counts when compared with the NC group (*P* < 0.01, Figure 2A). However, BALFs from the PYC-treated groups (15 and 30 mg/kg) had significantly lower total cell counts when compared with the ASD group (both *P* < 0.01). When compared with the NC group, BALF from the ASD group had markedly higher numbers of neutrophils (Figure 2B, *P* < 0.01). However, when compared with the ASD group, the BALFs of the PYC-treated groups had lower levels of inflammatory cells, with the group treated with PYC at 30 mg/kg exhibiting a significant difference (*P* < 0.01). Additionally, when compared with the NC group, BALF from the ASD group had markedly higher macrophage numbers (*P* < 0.01, Figure 2C). In contrast, the BALF from the PTC-treated groups (15 and 30 mg/kg) exhibited significantly lower macrophage numbers when compared with the ASD group (*P* < 0.01 for both groups).

### Effects of PYC on the release of inflammatory cytokines in the ASD-induced model of pulmonary inflammation

**Figure 3:**
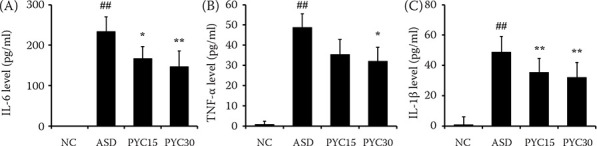
Effects of PYC on the levels of inflammatory cytokines in ASD-exposed mice (A) IL-6, (B) TNF-α, (C) IL-1β ASD = ASD intranasal instillation + PBS treatment; NC = PBS intranasal instillation + PBS treatment; PYC 15 and 30 = ASD intranasal instillation + PYC treatment at 15 mg/kg and 30 mg/kg, respectively Data are presented as mean ± SD (*n* = 5); ^##^*P* < 0.01 vs NC, *^,^**P < 0.05 and < 0.01 (vs ASD), respectively

When compared with the control group, the ASD group had markedly higher levels of IL-6 (233.5 ± 36.9 pg/ml, *P* < 0.01, Figure 3A). However, when compared with the ASD group, the PYC-treated groups exhibited significantly lower IL-6 levels (166.5 ± 30.3 pg/ml for the 15 mg/kg group, *P* < 0.05 and 146.4 ± 39.3 pg/ml for the 30 mg/kg group, *P* < 0.01). In addition, when compared with the NC group, the ASD group had markedly higher TNF-α levels (48.8 ± 10.2 pg/ml, *P* < 0.01, Figure 3B). Moreover, treatment with PYC at 30 mg/kg significantly reduced the levels of IL-6 when compared with the ASD group (32.1 ± 9.9 pg/ml, *P* < 0.05), but 15 mg/kg of PYC did not significantly reduce IL-6 levels (35.4 ± 9.2 pg/ml). Similarly, markedly elevated levels of IL-1β levels were observed in the ASD group when compared with the control group (54.8 ± 6.7 pg/ml, *P* < 0.01, Figure 3C). In contrast, the PYC-treated groups exhibited significantly lower levels of IL-1β when compared with the ASD group (32.3 ± 7.4 pg/ml, *P* < 0.01 for the 15 mg/kg group, and 28.9 ± 6.9 pg/ml, *P* < 0.01 for the 30 mg/kg group).

### Effects of PYC on inflammatory responses in the ASD-induced model of pulmonary inflammation

**Figure 4:**
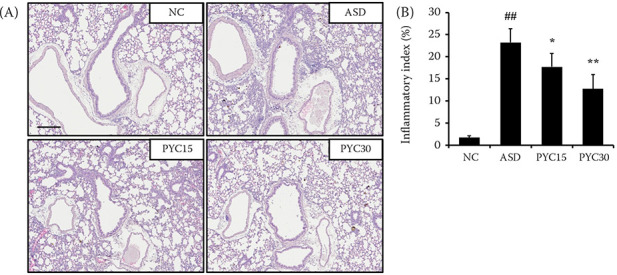
Effects of LTE on inflammatory responses in the lung tissue of ASD-exposed mice (A) Representative H&E image. (B) inflammatory index ASD = ASD intranasal instillation + PBS treatment; NC = PBS intranasal instillation + PBS treatment; PYC 15 and 30 = ASD intranasal instillation + PYC treatment at 15 mg/kg and 30 mg/kg, respectively Scale bar: 100 μm; Data are presented as mean ± SD (*n* = 5); ^##^*P* < 0.01 vs Con, *^,^**P < 0.05 and < 0.01 (vs ASD), respectively

Compared with the control group, the ASD group exhibited extensive inflammatory infiltration into the lung tissue (Figure 4A). However, the lung tissue of PYC-treated groups exhibited lower levels of inflammatory responses when compared with the ASD group. Quantitative analysis of inflammatory responses revealed that the ASD group had a markedly higher inflammatory index when compared with the NC group (23.2 ± 3.1%, *P* < 0.01, Figure 4B). In contrast, the PYC-treated groups had significantly lower inflammatory indexes when compared with the ASD group (17.7 ± 3.0%, *P* < 0.05 for the 15 mg/kg group and 12.7 ± 3.2%, *P* < 0.01 for the 30 mg/kg group).

### Effects of PYC on MMP-9 expression in the lung tissue of the ASD-induced model of pulmonary inflammation

**Figure 5:**
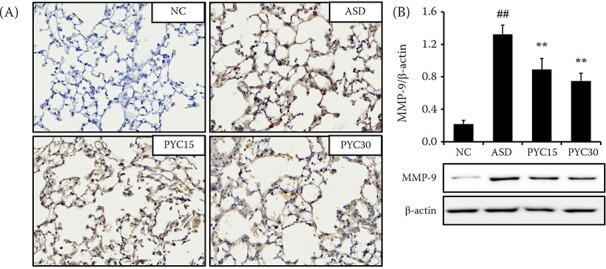
Effects of LTE on MMP-9 expression in ASD-exposed mice. (A) Representative IHC image. (B) densitometric protein-expression levels ASD = ASD intranasal instillation + PBS treatment; NC = PBS intranasal instillation + PBS treatment; PYC 15 and 30 = ASD intranasal instillation + PYC treatment at 15 mg/kg and 30 mg/kg, respectively Data are presented as mean ± SD (*n* = 5); ^##^*P* < 0.01, vs Con; *^,^**P < 0.05 and < 0.01 (vs ASD), respectively

When compared with the NC group, ASD exposure elevated MMP-9 expression in lung tissue (Figure 5A). However, when compared with the ASD group, the PYC groups exhibited a dose-dependent reduction in the level of MMP-9 expression in the lung tissue. Western blot analysis revealed that the ASD group had markedly higher MMP-9 expression than the NC group (1.3 ± 0.2, *P* < 0.01). In contrast, the PYC groups had significantly lower MMP-9 levels when compared with the ASD group (0.9 ± 0.1, *P* < 0.01 for the 15 mg/kg group and 0.7 ± 0.1, *P* < 0.01, for the 30 mg/kg group).

### Effect of PYC on the expression of Nrf2 and 8-OHdG in the lung tissue of the ASD-induced model of pulmonary inflammation

**Figure 6:**
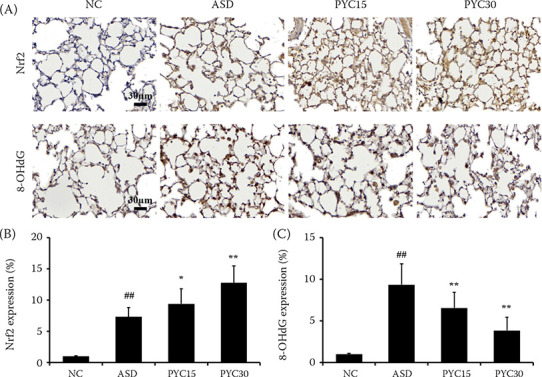
Effects of PYC on the expression of Nrf2 and 8-OHdG in ASD-exposed mice (A) Representative IHC image. (B) quantitative analysis of Nrf2 expression. (C) quantitative analysis of 8-OHdG expression ASD = ASD intranasal instillation + PBS treatment; NC = PBS intranasal instillation + PBS treatment; PYC 15 and 30 = ASD intranasal instillation + PYC treatment at 15 mg/kg and 30 mg/kg, respectively Data are presented as mean ± SD (*n* = 5); ^##^*P* < 0.01, vs Con; *^,^**P < 0.05 and < 0.01 (vs ASD), respectively

When compared with the NC group, ASD exposure elevated Nrf2 expression in lung tissue (Figure 6A,B).

However, when compared with the ASD group, the PYC groups exhibited a dose-dependent elevation in the level of Nrf2 expression in the lung tissue. 8-OHdG expression was markedly increased in the ASD group compared with the NC group (Figure 6A,C). However, the PYC groups exhibited a significant reduction in 8-OHdG expression in comparison to the ASD group.

## DISCUSSION

The increasing levels of air pollution have increased the prevalence of various diseases. ASD, which affects Northeast Asia during spring and autumn, is associated with several diseases in China, Japan, and Korea, including respiratory, ocular, and skin diseases (Sadakane et al. 2022). Moreover, rapid industrialization is exacerbating the threat posed by ASD to human and animal health because of the presence of harmful substances, including nanoscale materials, heavy metals, organic chemicals, and allergens (Sadakane et al. 2019; Shin et al. 2022). To minimise the risk of ASD, the country is putting various measures in place, such as ASD warnings and recommendations to wear masks. However, preparedness to treat the harmful effects of ASD on the respiratory system is limited. In this study, we investigated the therapeutic effects of PYC on ASD-induced pulmonary inflammation. We find that PYC significantly decreased inflammatory cell counts in the BALF of ASD-exposed mice and reduced the levels of inflammatory cytokines. These observations were supported by histological examination which revealed that PYC markedly decreased ASD-induced inflammatory cell infiltration into lung tissue. Moreover, PYC significantly decreased MMP-9 expression levels in the lungs of ASD-exposed mice.

Because ASD accumulates several harmful materials during its travel over long distances, it induces inflammatory responses in lung tissue or aggravates underlying respiratory diseases (Nakao et al. 2018). ASD exposure is reported to elevate the production of inflammatory cytokines, which accelerate inflammatory cell infiltration into lung tissue, resulting in pulmonary inflammation (Park et al. 2021). In this study, we found that exposure to ASD elevated the levels of IL-1β, IL-6, and TNF-α. Interestingly, treatment with PYC significantly suppressed ASD-induced inflammatory cytokine production, which was accompanied by reduced inflammatory cell counts in the BALFs of ASD-exposed mice. These results indicate that PYC effectively suppressed ASD-driven pulmonary inflammation.

MMP-9, a proteolytic enzyme involved in the degradation of the extracellular matrix (Cabral-Pacheco et al. 2020), is associated with the development of various diseases, including arthritis, cancer, and inflammatory disorders (Hannocks et al. 2019; Mondal et al. 2020; Bassiouni et al. 2021). MMP-9 is reported to stimulate neutrophil migration into lesions and to induce the production of inflammatory cytokines, chemokines, and reactive oxygen species, which eventually disrupts the normal tissue structure, aggravating inflammatory responses (Ko et al. 2019). In the respiratory tract, MMP-9 elevation damages the alveolar structure via the degradation of collagen and gelatin components of the extracellular matrix and produces inflammatory cytokines, which drives pulmonary inflammation or aggravates underlying diseases, such as asthma and chronic obstructive pulmonary disease (Shin et al. 2017; Lim et al. 2022). Exposure to ASD not only induces the production of inflammatory cytokines but also elevates MMP-9 expression (Shimada et al. 2015). Here, we find that the administration of PYC effectively suppressed ASD-induced MMP-9 expression and reduced the level of inflammatory cytokines, indicating that the therapeutic effects of PYC against ASD-induced pulmonary inflammation involve the suppression of MMP-9 expression.

Oxidative stress is an important mediator in the pathogenesis and development of various diseases. To protect themselves from oxidative stress, organisms have various antioxidant systems. Of antioxidant systems, Nrf2 plays a key regulator in the reduction of oxidative stress (Jung et al. 2020). Nrf2 expression is markedly increased by the production of reactive oxygen species and translocated into the nucleus, which leads to the elevation of antioxidant proteins (Sakurai et al. 2018). In this study, ASD exposure induced marked oxidative damage to lung tissue evidenced by increases in 8-OHdG expression. However, PYC treatment significantly reduced the expression of 8-OHdG in lung tissue, which was accompanied by the enhancement of Nrf2 expression. These results indicated that PYC effectively decreases oxidative damage induced by ASD exposure.

PYC, a standardised French maritime pine bark extract, contains phenolic acids and procyanidins. Because of its antioxidant, anti-inflammatory, and anticancer properties, PYC is used as a food or dietary supplement to control various pathological conditions, such as hepatotoxicity, cardiotoxicity, and nephrotoxicity. PYC is also reported to have therapeutic effects against respiratory diseases like asthma and chronic obstructive pulmonary disease (Shin et al. 2013; Ko et al. 2017). For instance, in ovalbumin-induced asthmatic mice, PYC is reported to inhibit MMP-9 expression and in people, it reduces MMP-9 secretion into the plasma (Grimm et al. 2006; Shin et al. 2013), which is consistent with our findings.

In this study, we did not use a group of drugs exhibiting therapeutic effects in ASD-induced inflammation. This is because the drug to be applied to pulmonary inflammation caused by ASD exposure has not yet been determined. However, since steroids are used to compare drugs in various pulmonary inflammation models, steroids are considered to be suitable for ASD-induced pulmonary inflammation models. In addition, the steroids were commonly used for comparing the therapeutic effects of PYC in various experiments (Gunel et al. 2016; Unsal et al. 2018).

Taken together, our data show that PYC administration effectively inhibited ASD-induced pulmonary inflammation and suppressed MMP-9 expression, highlighting PYC as a potential therapeutic agent for treating ASD-driven pulmonary inflammation.
