# Erratum

**DOI:** 10.17221/97/2024-VETMED

**Published:** 2024-11-29

**Authors:** SW Pak, SJ Lee, WI Kim, YG Yang, YK Cho, JS Kim, TW Kim, JW Ko, JC Kim, SH Kim, IS Shin

To the published article: Pak SW, Lee SJ, Kim WI, Yang YG, Cho YK, Kim JS, Kim TW, Ko JW, Kim JC, Kim SH, Shin IS (2024): **The effects of Pycnogenol, a pine bark extract on pulmonary inflammation by Asian sand dust in mice**. Vet Med-Czech 69, 8–17 (doi: 10.17221/77/2023-VETMED).

A funding source has been added, page: 8.

The editorial team apologises for any inconvenience that it may have caused.

Original title page data:

”No funding.”

Corrected title page data:

“Supported by the national Research Foundation of Korea granted by Korea Government (NRF-2020R1A4A1019395 and NRF-2022R1A2C1010628).”

